# A novel aurone RNA CAG binder inhibits the huntingtin RNA–protein interaction†

**DOI:** 10.1039/d4md00403e

**Published:** 2024-07-17

**Authors:** Giovanna Ballarin, Maddalena Biasiotto, Annika Reisbitzer, Marlen Hegels, Michael Bolte, Sybille Krauß, Daria V. Berdnikova

**Affiliations:** a University of Padova, School of Pharmaceutical Sciences via Marzolo 5 35131 Padova Italy; b Institut für Biologie, Universität Siegen Adolf-Reichwein-Str. 2 57076 Siegen Germany; c Organische Chemie II, Universität Siegen Adolf-Reichwein-Str. 2 57076 Siegen Germany berdnikova@chemie-bio.uni-siegen.de; d Institut für Anorganische Chemie, J.-W.-Goethe-Universität Max-von-Laue-Str. 7 60438 Frankfurt-am-Main Germany

## Abstract

Huntington's disease (HD) is a devastating, incurable condition whose pathophysiological mechanism relies on mutant RNA CAG repeat expansions. Aberrant recruitment of RNA-binding proteins by mutant CAG hairpins contributes to the progress of neurodegeneration. In this work, we identified a novel binder based on an aurone scaffold that reduces the level of binding of HTT mRNA to the MID1 protein *in vitro*. The obtained results introduce aurones as a novel platform for the design of functional ligands for disease-related RNA sequences.

## Abstract 2

A novel aurone binder for CAG RNA repeats has been identified from a library of twenty-eight compounds. The ligand inhibits toxic RNA–protein interactions in the Huntington's disease model.

## Graphical abstract

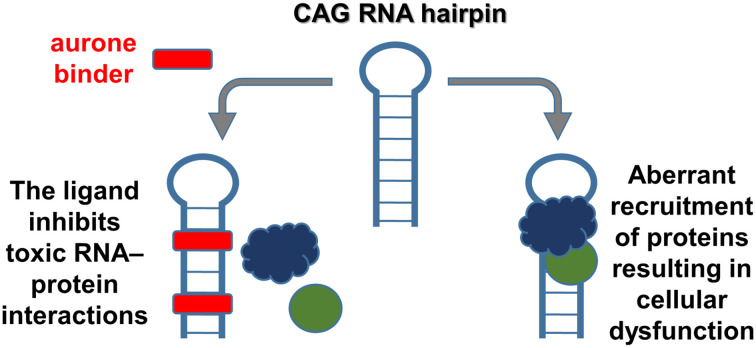

## Introduction

Short tandem repeats along with other repetitive sequences comprise a substantial fraction of the human genome.^1^ Being polymorphic and susceptible to mutations, short tandem repeats can elongate yielding repeat expansions, which become toxic after crossing a certain length threshold.^2–5^ These repeat expansions, scattered throughout the human genome, can lead to more than 40 severe disorders, the majority of which affect the nervous system and are currently incurable.^6,7^ Most often, repeat expansion disorders are caused by the expansion of CXG trinucleotide sequences. Among them are Huntington's disease (HD), myotonic dystrophy type 1 (DM1), a range of spinocerebellar ataxias, Fuchs corneal dystrophy and others.^6,7^

One of the well-known types of CAG repeat expansion disorders is Huntington's disease (HD), which leads to the progressive degeneration of brain nerve cells.^8^ The pathophysiological mechanism of HD is based on the expanded CAG repeat formed within exon 1 of the huntingtin (HTT) mRNA. Since the mutant repeat is located within a coding region, it becomes translated into a toxic polyglutamine-containing HTT protein that causes neurodegeneration and other consequences.^9^ The CAG repeats additionally contribute to the development of HD through another mechanism. The CAG repeat expansions significantly alter the RNA structure because the trinucleotide expansions fold into aberrant hairpins, which never form in a normal RNA. The aberrant hairpins can recruit RNA-binding proteins by providing additional binding sites that are not characteristic of a healthy RNA. Particularly, in the case of HD, proteins involved in translation induction and splice factors can become bound by this mechanism resulting in the loss of regular functions of these proteins and their potentially abnormal behavior.^10–14^

Understanding of biomolecular mechanisms underlying the development of the repeat expansion disorders paves a way towards potential therapeutical strategies for diagnostics and treatment. One of the strategies relies on selective interactions of the CAG repeat expansions with small organic molecules, which prevents the formation of toxic RNA–protein complexes. Although a range of small organic compounds that target CXG repeat expansion RNAs have been designed,^15–17^ there is still an urgent need in the development of RNA binders that selectively interact with the HD-associated CAG RNAs and block their aberrant biological functions. Along these lines, we became interested in aurones^18–20^ – a family of natural and synthetic flavonoids – as potential scaffolds for the design of binders for the CAG RNA. Depending on the substitution pattern, aurone derivatives demonstrate various biological activities, in general, upon selective interactions with proteins (enzymes).^18–20^ Some aurones possess antioxidant activity^21^ and antibacterial properties.^22^ At the same time, interactions of aurones with nucleic acids have been scarcely studied, so far.^18^ There are reports on the DNA-scission activity of some aurone derivatives^23^ and fluorescent DNA staining with aurones.^24^ However, to the best of our knowledge, the RNA-binding properties of aurones have not been described, so far.

Herein, we report a novel aurone derivative that selectively binds to the HD-associated CAG repeat expansion RNA and inhibits the RNA–protein interaction in an RNA pull-down assay *in vitro*.

## Results and discussion

### Synthesis

**Chart 1:**
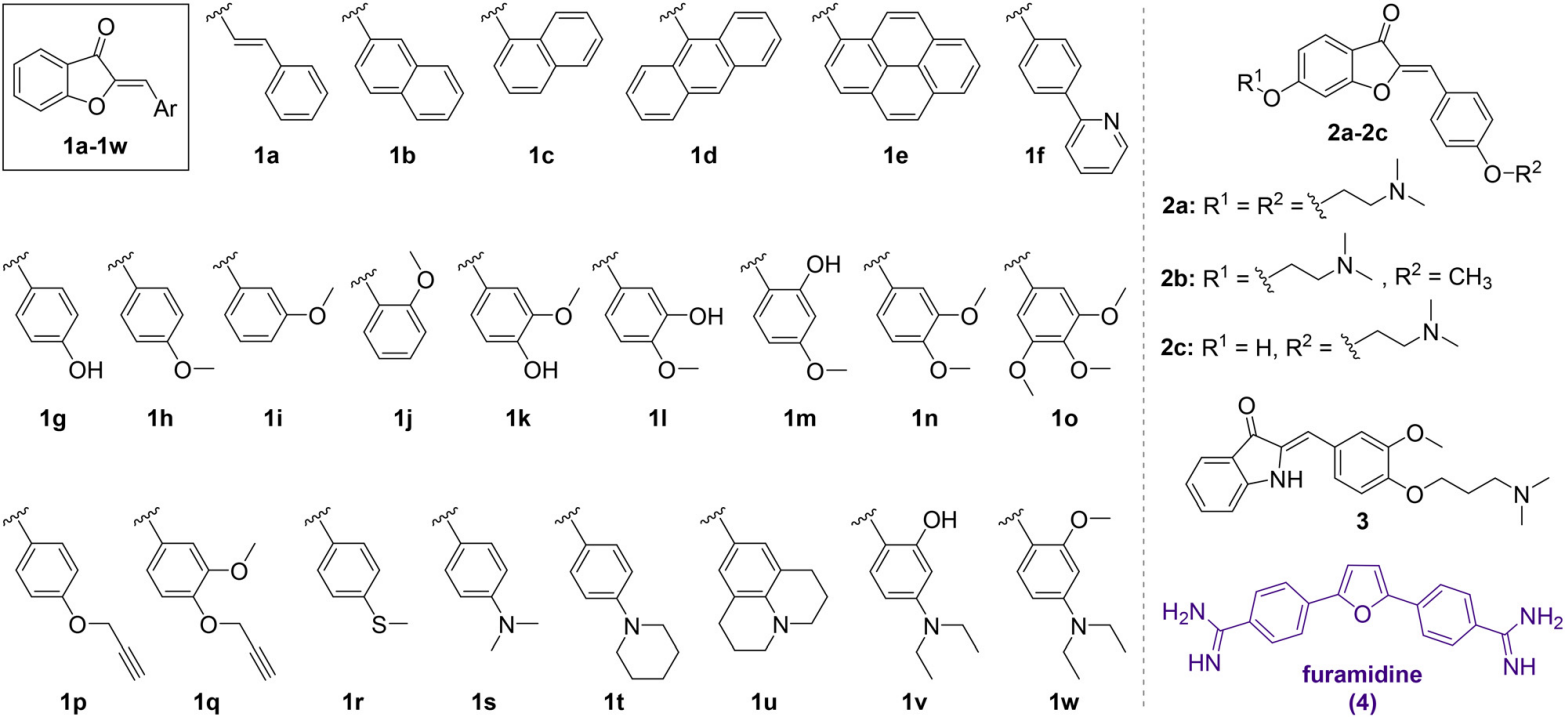
Chemical structures of aurone derivatives 1a–1w and 2a–2c and aza-aurone (hemiindigo) 3 used for the screening and the structure of the known CAG RNA binder furamidine (4).

**Fig. 1:**
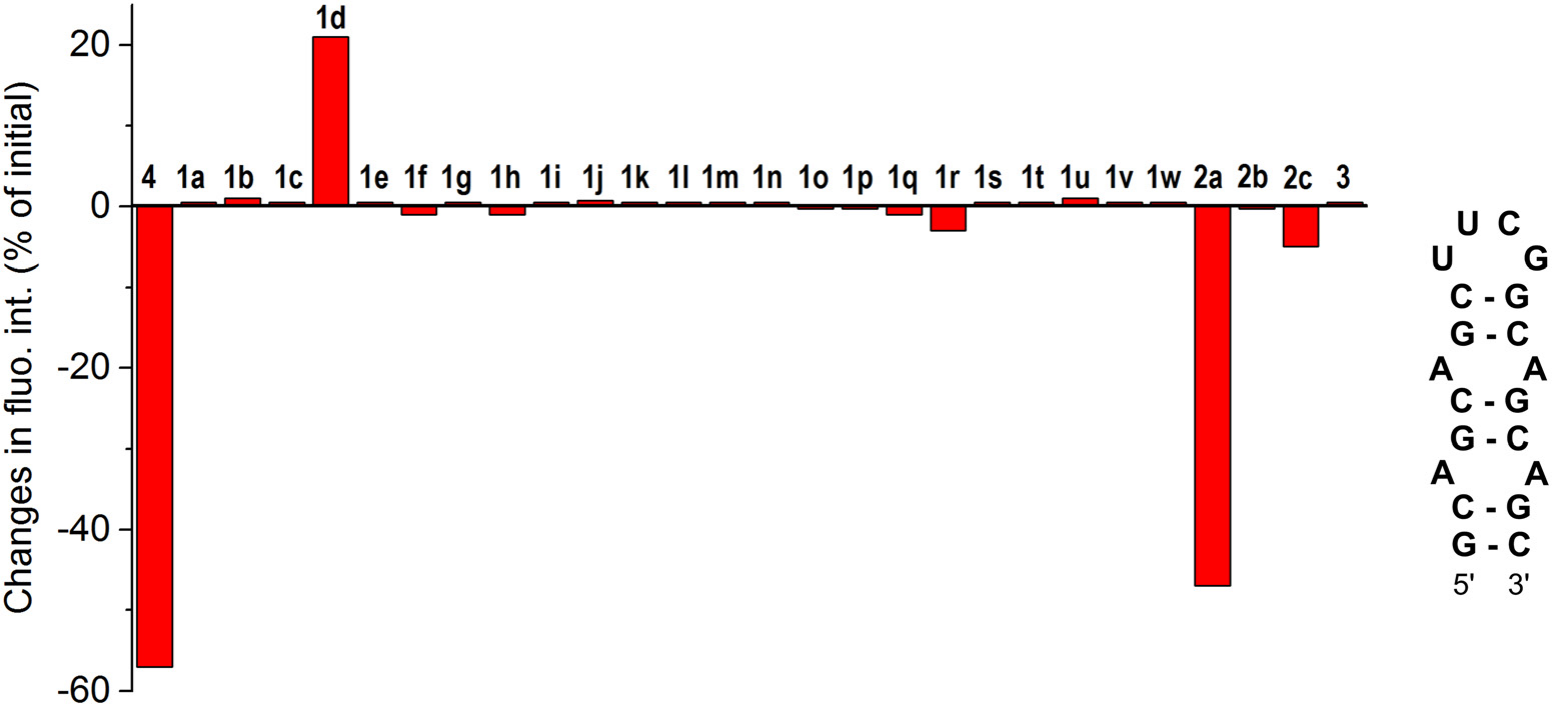
Changes in the fluorescence intensity (in % of the initial intensity) of aurones 1a–1w and 2a–2c, aza-aurone 3 and furamidine (4) (*c*_lig_ = 5 μM) in the presence of 1 equiv. of the 5′-GCAGCAGCUUCGGCAGCAGC-3′ oligonucleotide in a buffer (pH = 7); positive values indicate fluorescence light-up in the presence of RNA and negative values indicate fluorescence quenching in the presence of RNA. The fluorescence output of each ligand was measured at three different excitation wavelengths: *λ*_ex_ = 350, 400 and 450 nm. The most reliable fluorescence output was obtained upon excitation at *λ*_ex_ = 350 nm for most compounds except for 1d (*λ*_ex_ = 450 nm) and 2b (*λ*_ex_ = 400 nm). To ensure the reproducibility, each measurement was repeated at least three times, and the repeat experiments gave values within 20%.

To initially assess the RNA binding potential of aurone ligands and identify the possible hits, a library of twenty-six aurone derivatives 1a–1w and 2a–2c bearing different substituents and one aza-aurone (hemiindigo) derivative 3 (Chart 1) were screened against a short RNA oligonucleotide 5′-GCAGCAGCUUCGGCAGCAGC-3′ comprising two CAG repeats (Fig. 1).^25^ Known compounds 1a–1e, 1g–1k, 1m–1o, 1r–1u, 1w, 2a–2c and 3 were synthesized in our lab earlier and characterized by comparison with the literature data for melting point values and NMR spectroscopy.^21,22,24,26–34^ Novel aurone derivatives 1f, 1l, 1p, 1q, and 1v were obtained in this work for the first time and fully characterized by 1D and 2D NMR spectroscopy, mass spectrometry and elemental analysis (ESI†). For compounds 1f and 1w, single-crystal X-ray analysis data were provided for the first time (ESI,† CCDC deposition numbers: 2335536 and 2335537).

### Well-plate screening

The screening of 1a–1w, 2a–2c and 3 against the CAG RNA oligonucleotide was performed using a well plate and the fluorescence output of each ligand was measured without RNA and in the presence of one equivalent of the RNA oligonucleotide at three different excitation wavelengths (*λ*_ex_ = 350, 400 and 450 nm). The known CAG RNA binder furamidine (4)^35^ was included in the screening as a reference compound. The results of the screening are provided in Fig. 1. As can be seen, most of the compounds demonstrated negligible changes in the fluorescence response upon the addition of RNA, indicating an absent or a very weak interaction with the nucleic acid. Notably, the previously reported HIV RNA binder 3^33^ did not associate with CAG RNA. Only for three derivatives out of the twenty-eight screened ones, the changes in fluorescence in the presence of RNA were pronounced. Thus, as expected, the known CAG RNA binder furamidine (4) showed significant fluorescence quenching (57% of the initial intensity). The fluorescence of aurone 2a bearing two alkylamino chains was also remarkably quenched (47% of the initial intensity) upon the addition of the CAG RNA oligonucleotide. Notably, aurones 2b and 2c having only a single alkylamino chain showed small changes in the fluorescence intensity pointing out a crucial binding role of the second alkylamino pendant in 2a. The aurone derivative 1d comprising an anthracenyl moiety demonstrated a moderate fluorescence light-up effect (21% of the initial intensity) in the presence of the CAG RNA oligonucleotide. Due to significant changes in the fluorescence output in the presence of the CAG RNA oligonucleotide, aurone derivatives 1d and 2a were selected for further analysis.

### Selectivity studies

**Fig. 2:**
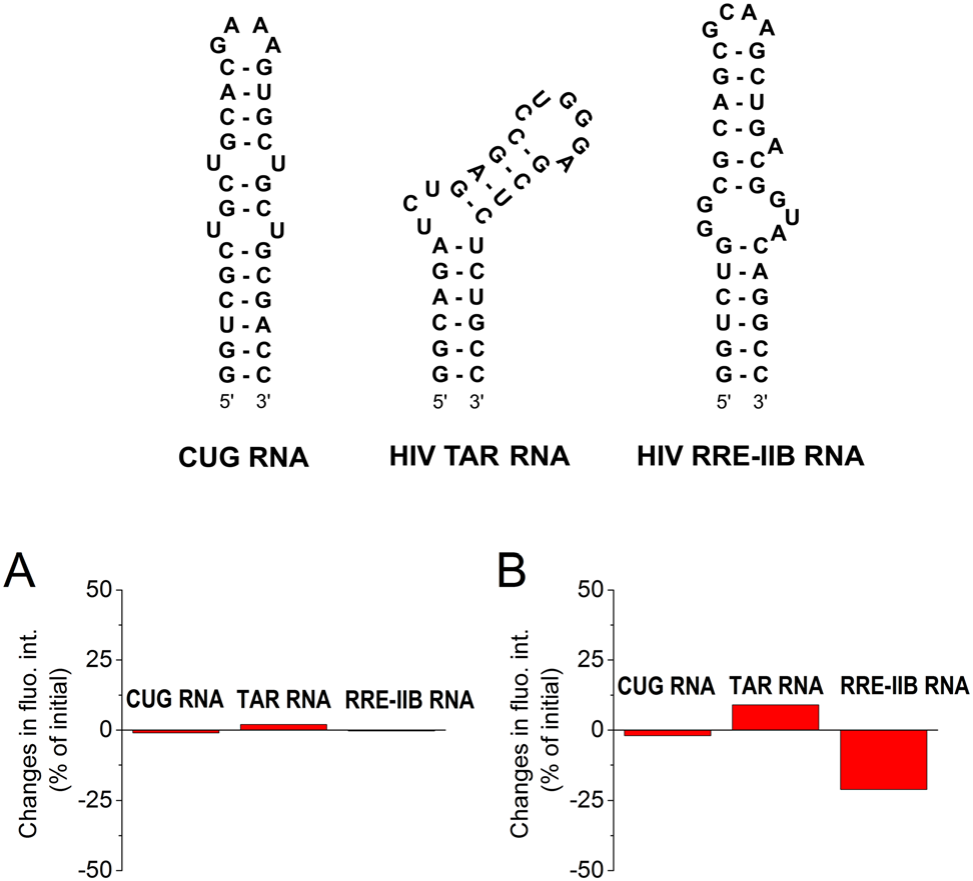
Structures of the CUG RNA, HIV-1 TAR RNA and HIV-1 RRE-IIB RNA oligonucleotides used in this study and changes in the fluorescence intensity (in% of the initial intensity) of aurones (A) 2a and (B) 1d (*c*_lig_ = 5 μM) in the presence of 1 equiv. of each RNA oligonucleotide in a buffer (pH = 7); positive values indicate fluorescence light-up in the presence of RNA and negative values indicate fluorescence quenching in the presence of RNA. The fluorescence output was measured upon excitation at *λ*_ex_ = 350 nm for 2a and at *λ*_ex_ = 450 nm for 1d. To ensure the reproducibility, each measurement was repeated at least three times, and the repeat experiments gave values within 20%.

To assess the selectivity of interactions of aurones 1d and 2a with CAG RNA, additional well-plate screening was performed using alternative RNA substrates, namely the CUG RNA motif associated with myotonic dystrophy type 1 (DM1) and the regulatory elements of human immunodeficiency virus type 1 (HIV-1) genome RNA – the transactivation response element (TAR) and the stem IIB of the Rev response element (RRE-IIB) (Fig. 2).^36,37^ The HIV-1 TAR and RRE-IIB RNAs were chosen for this study because their structure allows to test several possible binding modes between the small ligands and RNA, including the stem intercalation, bulge binding and loop binding. In principle, stem intercalation can also take place in the case of the used CUG oligomer comprising an elongated double-helix region (Fig. 2). Like in the case of the CAG RNA oligonucleotide, the fluorescence of aurones 1d and 2a was recorded on the well-plate without RNA and in the presence of 1 equivalent of the corresponding oligonucleotides (Fig. 2). It was found that the addition of these RNA oligonucleotides produced almost no effect on the fluorescence of compound 2a indicating an absent or very weak interaction. For derivative 1d, almost no interaction was detected with the CUG RNA. However, the changes in the fluorescence of 1d in the presence of the TAR RNA (light-up) and RRE-IIB RNA (quenching) were pronounced and, therefore, indicative of binding. Therefore, within the tested RNA sequences, aurone 2a showed clear selectivity towards the CAG trinucleotide repeat motif. At the same time, compound 1d was much less selective towards various RNA sequences (noticeable interactions with HIV TAR and RRE-IIB RNA), although it obviously showed preference for the CAG RNA in comparison to the CUG RNA. Notably, in the case of compound 2a, two alkylamino substituents played a crucial role in the development of the RNA-binding properties and selectivity towards the CAG RNA. For comparison, the core scaffold of derivative 2a, namely 4-methoxyaurone (compound 1h, Fig. 1), did not interact with RNA. Moreover, the related compounds 2b and 2c bearing a single alkylamino substituent either at the coumaranone fragment (2b) or at the phenyl ring (2c) did not show a pronounced interaction with the CAG RNA (Fig. 1). A careful comparison can also be made with the aza-aurone derivative 3 bearing only one alkylamino tail: the presence of a single alkylamino substituent did not provide the affinity towards the CAG RNA (Fig. 1).

### Determination of the binding constant with the CAG RNA oligonucleotide

**Fig. 3:**
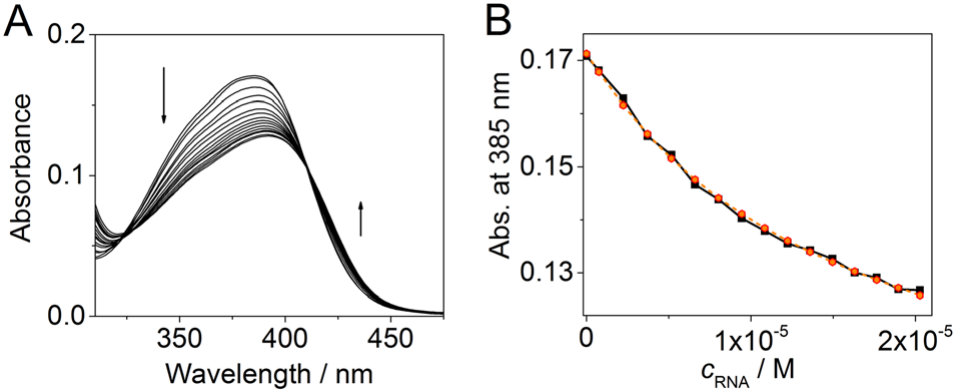
(A) Spectrophotometric titration of 2a with the 5′-GCAGCAGCUUCGGCAGCAGC-3′ RNA oligonucleotide (*c*_2_ = 5 μM and RNA/*c*_2_ = 0–4) and (B) binding isotherm, *i.e.* a plot of the absorbance of 2a*versus* concentration of RNA (*c*_RNA_), obtained from the photometric titration; black solid line: experimental data and orange dashed line: fit to the theoretical model.

To quantify the interaction of aurone derivatives with the CAG RNA oligonucleotide, spectrophotometric titration was performed (Fig. 3A). Thus, upon the addition of RNA, the absorption spectrum of ligand 2a showed a hypochromic effect along with a moderate red shift of the absorption maximum. During the titration, a clear isosbestic point at 407 nm was formed indicating a single dominating binding mode of 2a as well as homogeneous folding of the RNA motif providing preferentially a single type of the binding pocket. The analysis of the obtained binding isotherm (Fig. 3B) allowed to estimate the stoichiometry and the binding constant of the 2a–RNA complex. Thus, the preferential formation of the 1 : 1 ligand–RNA oligonucleotide complexes was observed (for details, see the ESI†). The association constant value is *K* = (1.4 ± 0.1) × 10^5^ M^−1^. For derivative 1d, the determination of the binding constant by spectrophotometric titration was not possible due to the aggregation of the compound upon the addition of RNA.

### RNA pull-down experiments

**Fig. 4:**
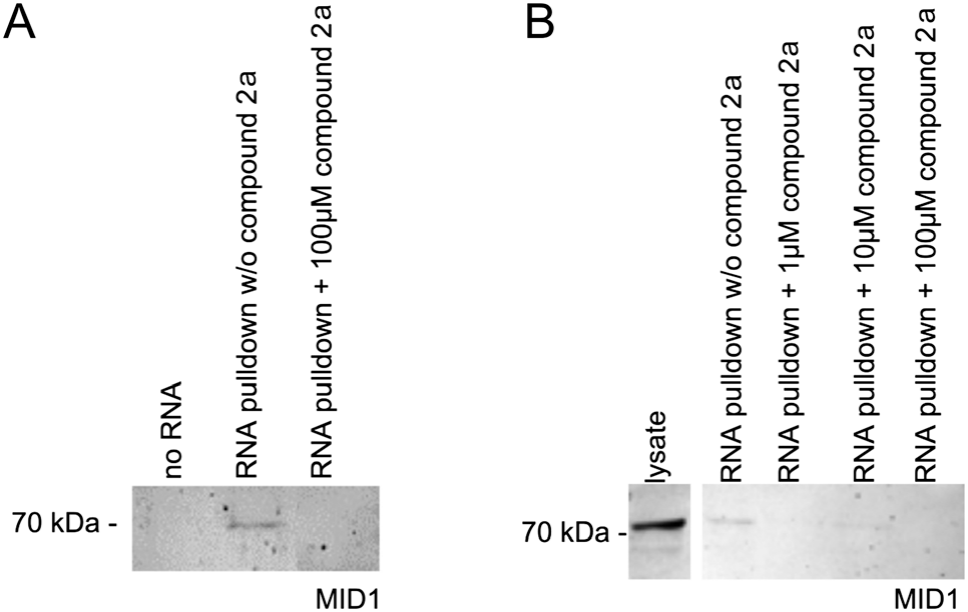
RNA–protein pull-down of MID1 with its target RNA HTT exon 1 in the absence (w/o compound 2a) or the presence of compound 2a. RNA-bound proteins were analyzed by western blot detecting MID1. (A) RNA–protein pull-down in the presence or the absence of compound 2a at a final concentration of 100 μM. A negative control that does not contain RNA was included (no RNA). The expected band of approx. 70 kDa was detected in the RNA pull-down without the compound in the cell lysate. (B) RNA–protein pull-down as described in (A) with different doses of compound 2a (final concentrations of 1 μM, 10 μM, and 100 μM).

As has been shown previously, the MID1 protein binds its target HTT mRNA at its CAG-repeat in a length-dependent manner.^11,12,35^ Therefore, we used HTT exon 1 transcripts containing the CAG-repeat region (for details, see the ESI†) to test if compounds 2a and 1d affect the binding between the MID1 protein and its target mRNA by performing RNA–protein pull-down assays. To perform this experiment, biotinylated RNA-oligos were incubated with cell extracts that contained the MID1 protein in the presence or the absence of 2a and 1d. The RNA–protein complexes were then isolated using streptavidin beads, and the RNA-bound proteins were analyzed by western blot detecting MID1 (Fig. 4). As a negative control, an experiment without RNA was performed. As expected, MID1 was detected in the samples without compound 2a (positive control). At the same time, in the presence of 2a, the binding of MID1 to HTT RNA was suppressed (Fig. 4A). The dose-dependence assay (Fig. 4B) showed that aurone 2a provided the inhibition of the RNA–MID1 interactions at all tested doses (final concentrations from 1 μM to 100 μM). The pull-down assay with compound 1d did not reveal an inhibiting effect on the RNA–protein interactions (Fig. S1, ESI†). The possible reason for this is the low selectivity of 1d towards the CAG RNA motif (*vide supra*).

## Conclusions

In summary, we have identified novel CAG RNA binder 2a that inhibits the toxic RNA–MID1 protein interaction *in vitro* in the Huntington's disease model. To the best of our knowledge, this is the first example of an RNA binder based on an aurone scaffold, which, therefore, provides a proof-of-principle for the application of aurone flavonoids as a platform for the design of RNA-targeting ligands.

## Data availability

The data supporting this article have been included as part of the ESI.† Crystallographic data for 1f and 1w have been deposited at the CCDC under deposition numbers 2335536 and 2335537.

## Author contributions

D. V. B. and S. K. conceived and designed the experiments. G. B., M. B. (Maddalena Biasiotto), A. R., M. H. and D. V. B. performed the experiments. M. B. (Michael Bolte) carried out the single-crystal X-ray analysis. D. V. B. and S. K. performed the supervision, analysed the data and wrote the manuscript.

## Conflicts of interest

There are no conflicts to declare.

## Supplementary Material

MD-015-D4MD00403E-s001

MD-015-D4MD00403E-s002
