# Supplementary material for: A novel aurone RNA CAG binder inhibits the huntingtin RNA–protein interaction
Source: RSC Med Chem. 2024 Jul 17;15(9):3092–6. doi: 10.1039/d4md00403e (PMC11411626; doi:10.1039/d4md00403e)
Supplement: MD-015-D4MD00403E-s001 [file MD-015-D4MD00403E-s001.pdf]

Electronic Supplementary Material (ESI) for RSC Medicinal Chemistry.
This journal is © The Royal Society of Chemistry 2024

Supporting Information

A novel aurone RNA CAG binder inhibits the Huntingtin RNA–protein interaction

Giovanna Ballarin,[a,b,c,‡] Maddalena Biasiotto,[a,b,c,‡] Annika Reisbitzer,[b] Marlen Hegels,[c] Michael Bolte,[d] Sybille Krauß [b] and Daria V. Berdnikova*[c]

Table of Contents:

1. Materials and equipment S2

2. Synthesis S3

3. Calculation of the binding constants for **2a**–RNA complex S6

4. Biological studies S7

5. NMR data S8

6. Single-crystal X-ray crystallography data S14

7. References S16

1. Materials and equipment

Reagents and solvents for synthesis were obtained from commercial sources (Acros, Merck, Fischer) and used as received. Reactions were monitored on POLYGRAM® SIL G/UV_{254} (Macherey-Nagel) TLC plates with detection by UV light irradiation (254 nm or 366 nm).

^{1}H NMR and ^{13}C NMR spectra were recorded on *JEOL ECZ 500* spectrometers at 25 °C using 5-mm tubes. Chemical shifts were determined with accuracy of 0.01 ppm and 0.1 ppm for ^{1}H and ^{13}C spectra, respectively, and are given relative to the residual signal of the solvent that was used as internal standard (DMSO-d_6: δ_H = 2.50 ppm, δ_C = 39.5 ppm; acetone-d_6: δ_H = 2.05 ppm, δ_C = 29.9 ppm;). Spin–spin coupling constants for the proton spectra were determined with accuracy of 0.2 Hz. The proton NMR signal assignments were performed using COSY 2D NMR technique. The carbon NMR signal assignments were performed by means of HSQC and HMBC 2D NMR techniques. Since aurones lack characteristic protons in the heterocyclic ring, whose NOE correlation with the olefinic proton would allow for clear attribution of the double bond configuration, the identification of the *Z*-configuration of the novel compounds **1f**, **1l**, **1p**, **1q**, **1v** was performed using ^{13}C NMR shifts. Thus, the olefinic carbon atoms =C̲H of *Z*-aurones resonate at ~108–112 ppm [S1] that closely matches the chemical shifts of the C-10 carbons of derivatives **1f**, **1l**, **1p**, **1q**, **1v** (see below) confirming the *Z*-configuration.

Electrospray ionization (ESI) mass spectra were recorded on a *Finnigan LCQ Deca* mass-spectrometer (U=6 kV; working gas: Ar; auxiliary gas: N_2; temperature of the capillary: 200 °C).

Elemental analysis was performed with a *HEKAtech EUROEA* combustion analyser by Mr. Rochus Breuer (Universität Siegen, Organische Chemie I).

Melting points were measured with a *BÜCHI 545* (BÜCHI, Flawil, CH) melting point apparatus in open capillaries and are uncorrected.

Fluorescence spectra were measured on a Cary Eclipse two-beam spectrophotometer using a well plate for 96 samples (dots), solution volume per dot is 150 µL.

Electronic absorption spectra were measured on a Cary 100 Bio two-beam spectrophotometer.

Crystal structure determinations. Data for structures **1f** and **1w** were collected on a STOE IPDS II two-circle diffractometer with a Genix Microfocus tube with mirror optics using MoK_α radiation (λ = 0.71073 Å). The data were scaled using the frame scaling procedure in the *X-AREA* program system [S2]. The structures were solved by direct methods using the program *SHELXS* [S3] and refined against F^2 with full-matrix least-squares techniques using the program *SHELXL* [S3]. CCDC deposition numbers: 2335536 and 2335537.

2. Synthesis

Previously described aurones **1a–1e**, **1g–1k**, **1m–1o**, **1r–1u**, **1w**, **2a** and **3** were obtained and characterized according to the published data [21,22,24,26–33]. Novel synthetic approaches were used for the synthesis of previously reported compounds **2b** and **2c**, the products were characterized by comparison with the literature data for ^{1}H NMR and melting points [34].

General procedure for the synthesis of novel aurone derivatives 1f, 1l, 1p, 1q

Synthesis of novel aurone derivatives **1f**, **1l**, **1p**, **1q** was performed under basic conditions according to M. Varma et al. [30]: 3-coumaranon (134 mg, 1.00 mmol) and the corresponding arylaldehyde (1.25 mmol) were dissolved in DCM (3 mL), then neutral alumina (3.25 g) was added, and the mixture was vigorously stirred for 2–3 h at r. t. Then alumina was filtered off and washed with DCM (3 x 15 mL). The solution was collected and evaporated *in vacuo*. The crude products were purified by recrystallisation from EtOH.

(Z)-2-(4-(pyridin-2-yl)benzylidene)benzofuran-3(2H)-one **(1f)**

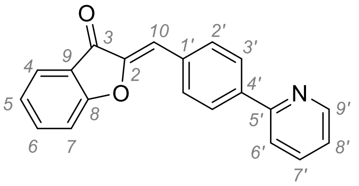

Yellow needles, yield 58% (174 mg, 0.58 mmol), m. p. 151–152 °C. ^{1}H NMR (500 MHz, DMSO-d_6, δ ppm, J Hz): 7.02 (s, 1H, H-10), 7.34 (td, 1H, H-5, J = 7.4, 0.8), 7.40 (ddd, 1H, H-8', J = 7.5, 4.7, 1.1), 7.61 (d, 1H, H-7, J = 8.9), 7.79–7.86 (m, 2H, H-4, H-6), 7.92 (td, 1H, H-7', J = 7.7, 1.8), 8.06 (dt, 1H, H-6', J = 8.0, 1.1), 8.12 (d, 2H, H-2', J = 8.4), 8.24 (d, 2H, H-3', J = 8.5), 8.71 (ddd, 1H, H-9', J = 4.7, 1.8, 0.9). ^{13}C NMR (126 MHz, DMSO-d_6, δ ppm): 111.7 (1C, C-10), 113.3 (1C, C-7), 120.6 (1C, C-6'), 120.8 (1C, C-9), 123.1 (1C, C-8'), 124.0 (1C, C-5), 124.3 (1C, C-4), 127.0 (2C, C-3'), 131.8 (2C, C-2'), 132.5 (1C, C-4'), 137.3 (1C, C-7'), 137.7 (1C, C-6), 139.8 (1C, C-1'), 146.6 (1C, C-2), 149.7 (1C, C-9'), 155.0 (1C, C-5'), 165.5 (1C, C-8), 183.6 (1C, C-3). Elem. anal. calcd. (%) for $C_{20}H_{13}NO_2$: C, 80.25; H, 4.38; N, 4.68; found: C, 80.34; H, 4.33; N, 4.77. ESI-MS **1f** in MeCN, *m/z*: calcd. 299.32; found 300.39 [**1f**+H]$^+$.

(Z)-2-(3-hydroxy-4-methoxybenzylidine)benzofuran-3(2H)-one **(1l)**

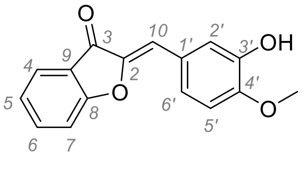

Thin yellow needles, yield 17% (46 mg, 0.17 mmol), m. p. 190–191 °C. ^{1}H NMR (500 MHz, DMSO-d_6, δ ppm, J Hz): 3.85 (s, 3H, OC<u>*H*</u>$_3$), 6.85 (s, 1H, H-10), 7.04 (d, 1H, H-5', J = 8.5), 7.31 (ddd, 1H, H-7, J = 8.3, 7.5, 0.9), 7.42 (dd, 1H, H-6', J = 8.8, 2.2), 7.52–7.55 (m, 2H, H-5, H-2'), 7.77–7.80 (m, 2H, H-4, H-6), 9.33 (s, 1H, O<u>*H*</u>). ^{13}C NMR (126 MHz, DMSO-d_6, δ ppm): 55.6 (1C, O<u>*C*</u>H$_3$), 112.2 (1C, C-5'), 113.1 (1C, C-5), 113.4 (1C, C-10), 117.7 (1C, C-2'), 121.2 (1C, C-9), 123.8 (1C, C-7), 124.2 (1C, C-4), 124.6 (1C, C-6'), 124.8 (1C, C-1'), 137.3 (1C, C-6), 145.1 (1C, C-2), 146.7 (1C, C-3'), 150.0 (1C, C-4'), 165.1 (1C, C-8), 183.2 (1C, C-3). Elem. anal. calcd. (%) for $C_{16}H_{12}O_4$: C, 71.64; H, 4.51; found: C, 71.43; H, 4.45. ESI-MS **1l** in MeCN, *m/z*: calcd. 268.26; found 267.19 [**1l**-H]$^+$.

(Z)-2-(4-(prop-2-yn-1-yloxy)benzylidene)benzofuran-3(2H)-one **(1p)**

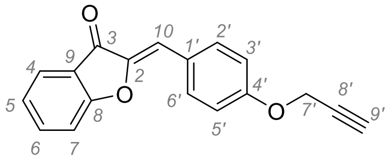

Yellow needles, yield 69% (190 mg, 0.69 mmol), ^{1}H NMR (500 MHz, DMSO-*d*$_6$, *δ* ppm, *J* Hz): 3.63 (t, 1H, H-9', *J* = 2.4), 4.90 (d, 2H, H-7', *J* = 2.4), 6.95 (s, 1H, H-10), 7.14 (d, 2H, H-3', H-5', *J* = 8.9), 7.32 (t, 1H, H-5, *J* = 7.7), 7.56 (d, 1H, H-7, *J* = 8.9), 7.80 (m, 2H, H-4, H-6), 7.99 (d, 2H, H-2', H-6', *J* = 8.9). ^{13}C NMR (126 MHz, DMSO-*d*$_6$, *δ* ppm): 55.6 (1C, C-7'), 78.6 (1C, C-9'), 78.8 (1C, C-8'), 112.4 (1C, C-10), 113.2 (1C, C-7), 115.5 (2C, C-3', C-5',), 121.1 (1C, C-9), 123.8 (1C, C-5), 124.2 (1C, C-4), 125.1 (1C, C-1'), 133.2 (2C, C-2', C-6'), 137.4 (1C, C-6), 145.3 (1 C, C-2), 158.7 (1C, C-4'), 165.2 (1C, C-8), 183.3 (1C, C-3). Elem. anal. calcd. (%) for $C_{18}H_{12}O_3$: C, 78.25; H, 4.38; found: C, 78.38; H, 4.45. ESI-MS **1p** in MeCN, *m/z*: calcd. 276.29; found 277.05 [**1p**+H]$^+$.

(Z)-2-(3-methoxy-4-(prop-2-yn-1-yloxy)benzylidene)benzofuran-3(2H)-one **(1q)**

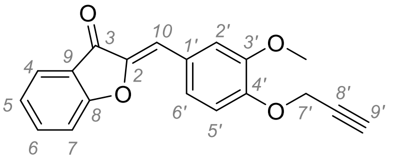

Yellow needles, yield 65% (199 mg, 0.65 mmol), m. p. 156–157 °C. ^{1}H NMR (500 MHz, DMSO-*d*$_6$, *δ* ppm, *J* Hz): 3.62 (t, 1H, H-9', *J* = 2.4), 3.86 (s, 3H, OC$\underline{H}_3$), 4.89 (d, 2H, H-7', *J* = 2.4), 6.94 (s, 1H, H-10), 7.17 (d, 1H, H-5', J = 8.3), 7.32 (t, 1H, H-7, *J* = 7.8), 7.57–7.59 (m, 1H, H-6'), 7.63–7.65 (m, 2H, H-5, H-2'), 7.78–7.81 (m, 2H, H-4, H-6). ^{13}C NMR (126 MHz, DMSO-*d*$_6$, *δ* ppm): 55.6 (1C, O$\underline{C}H_3$), 56.0 (1C, C-7'), 78.7 (1C, C-9'), 78.8 (1C, C-8'), 112.8 (1C, C-5'), 113.3 (1C, C-5), 113.8 (1C, C-10), 114.8 (1C, C-2'), 121.1 (1C, C-9), 123.8 (1C, C-7), 124.2 (1C, C-4), 125.1 (1C, C-6'), 125.5 (1C, C-1'), 137.3 (1C, C-6), 145.4 (1C, C-2), 148.4 (1C, C-3'), 149.1 (1C, C-4'), 165.2 (1C, C-8), 183.3 (1C, C-3). Elem. anal. calcd. (%) for $C_{19}H_{14}O_4$: C, 74.50; H, 4.61; found: C, 74.55; H, 4.58. ESI-MS **1q** in MeOH, *m/z*: calcd. 306.31; found 307.28 [**1q**+H]$^+$.

Synthesis of (*Z*)-2-(4-(diethylamino)-2-hydroxybenzylidene)benzofuran-3(2*H*)-one (1v)

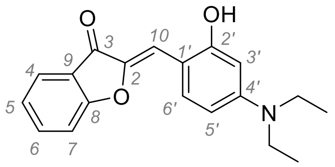

The synthesis was performed under acidic conditions according to Geissman et al. [S4]. A mixture of equimolecular amounts of 3-coumaranone (134 mg, 1.0 mmol) and 4-(diethylamino)salicylaldehyde (193 mg, 1.0 mmol) was dissolved in 5.0 mL of glacial acetic acid, then 0.3 mL of concentrated hydrochloric acid was added. The reaction was stirred at r. t. overnight. The mixture was poured into cold water to neutralize it, after which the product was collected and recrystallized from EtOH. Red solid, yield 18% (56 mg, 0.18 mmol); m. p. 186–187 °C; ^{1}H NMR (500 MHz, acetone-*d*$_6$, *δ* ppm, *J* Hz): 1.19 (t, 6H, C$\underline{H}_3$, *J* = 7.1), 3.45 (q, 4H, C$\underline{H}_2$, *J* = 7.1), 6.32 (s, 1H, H-3'), 6.43 (d, 1H, H-5', *J* = 9.0), 7.27 (t, 1H, H-6, *J* = 7.6), 7.44 (m, 2H, H-4, H-8), 7.72 (m, 2H, H-5, H-7), 8.17 (d, 1H, H-6', *J* = 9.0), 8.89 (s, 1H, O$\underline{H}$). ^{13}C NMR (125 MHz, acetone-*d*$_6$, *δ* ppm): 13.1 (1C, $\underline{C}H_3$), 45.2 (1C, $\underline{C}H_2$), 98.2 (1C, C-3'), 106.1 (1C, C-5'), 108.4 (1C, C-1'), 109.8 (1C, C-10), 113.7 (1C, C-7), 123.6 (1C, C-9), 123.9 (1C, C-5), 124.5 (1C, C-4), 134.2 (1C, C-6'), 136.5 (1C, C-6), 145.0 (1C, C-2), 152.1 (1C, C-4'), 160.4 (1C, C-2'), 165.7 (1C, C-8), 183.1 (1C, C-3). Elem. anal. calcd. (%) for $C_{19}H_{19}NO_3$: C, 73.77; H, 6.19; N, 4.53; found: C, 73.79; H, 6.14; N, 4.48. ESI-MS **1v** in MeCN, *m/z*: calcd. 309.36; found 310.20 [**1v**+H]$^+$.

Synthesis of (*Z*)-6-(2-(dimethylamino)ethoxy)-2-(4-methoxybenzylidene)benzofuran-3(2*H*)-one (2b)

Under exclusion of light, a suspension of (*Z*)-6-hydroxy-2-(4-methoxybenzylidene)benzofuran-3(2*H*)-one [S5] (130 mg, 0.49 mmol), anhydrous K_2CO_3 (201 mg, 1.46 mmol) and KI (2.42 mg, 0.15 mmol) in acetone (10 mL) was stirred at 60 °C for 1 h. Then (2-dimethylamino)-1-ethyl chloride hydrochloride (105 mg, 0.73 mmol) was added and the suspension was stirred at 60 °C for 7 h. The solvent was evaporated under reduced pressure and the residue was dissolved in CH_2Cl_2 (20 mL). The solution was extracted with an aq. solution of HCl (10%, 6 × 10 mL). The combined aqueous phases were adjusted to pH 12 by addition of an aq. solution of NaOH (10%), and cooled down with an ice bath. The formed precipitate was filtered off, further purification was not required. Yellow solid, yield 97% (160 mg, 0.47 mmol); m. p. 97–99 °C, (lit.: 93–94°C) [34]. ^{1}H NMR (500 MHz, $CDCl_3$, *δ* ppm, *J* Hz): 2.53 (s, 6H, -N($C\underline{H}_3$)$_2$), 2.96–3.02 (m, 2H, $C\underline{H}_2$-N), 3.87 (s, 3H, $OC\underline{H}_3$), 4.32 (t, 2H, -$OC\underline{H}_2$-, *J* = 5.4), 6.77 (dd, 1H, H-5, *J* = 8.5, 2.1), 6.80 (d, 1H, H-7, *J* = 2.1), 6.82 (s, 1H, $C\underline{H}$), 6.98 (d, 2H, Ar-*H*, *J* = 8.9), 7.71 (d, 1H, H-4, *J* = 8.5), 7.86 (m, 2H, Ar-*H*) (lit.: ref. [34]).

Synthesis of (*Z*)-2-(4-(2-(dimethylamino)ethoxy)benzylidene)-6-hydroxy-benzofuran-3(2*H*)-one (2c)

Adapted from Okombi et al. [S6]. To a solution of 6-hydroxy-3-coumaranone (92 mg, 0.62 mmol) in MeOH (4.2 mL), an aq. solution of KOH (50%, *w*/*w*, 0.92 mL) was added, followed by the addition of 4-(2-(dimethylamino)ethoxy)benzaldehyde (178 mg, 0.92 mmol) in MeOH (2 mL). The mixture was stirred at 60 °C for 1 h, and the solvent was removed under reduced pressure. The crude was diluted by water and the mixture was extracted with EtOAc (3 × 20 mL, 1 × 10 mL). Meanwhile, crystals of the product were formed in the aqueous phase, which were filtered off and washed with diethyl ether (2 × 5 mL), further purification was not required. Yellow solid, yield of 59% (117 mg, 0.36 mmol); m. p. 232–234 °C (lit.: 235–236 °C) [34]. ^{1}H NMR (500 MHz, DMSO-d_6, *δ* ppm, *J* Hz): 2.23 (s, 6H, $C\underline{H}_3$), 2.65 (t, 2H, $C\underline{H}_2$-N, *J* = 5.8), 4.12 (t, 2H, $C\underline{H}_2$-O, *J* = 5.8), 6.68 (dd, 1H, Ar-H, *J* = 8.4, 2.0), 6.75 (s, 1H, $C{=}C\underline{H}$), 7.06 (d, 2H, Ar-H, *J* = 9.0), 7.58 (d, 1H, Ar-H, *J* = 8.5), 7.90 (d, 2H, Ar-H, *J* = 8.7) (lit.: ref. [34]).

3. Calculation of the binding constants for 2a–RNA complex

The binding constant of the complex of compound **2a** with 5'-GCAGCAGCUUCGGCAGCAGC-3' oligonucleotide was determined using SpecFit/32 [Spectrum Software Associates, PMB 361, 197M Boston Post Road, West Marlborough, MA 01752, U.S.A.] from the binding isotherm obtained from the photometric titrations data. Due to the small size of the used RNA oligonucleotide and its well-defined secondary structure and tertiary folding, the ligand–oligonucleotide binding can be represented as a host–guest interaction, where one host molecule (RNA) provides one or several binding sites for the guest molecule (compound **2a**). The following equilibria were considered to occur simultaneously or separately:

Lig + RNA ⇔ Lig-RNA

2Lig + RNA ⇔ $(Lig)_2$-RNA

The best fit of the theoretical model to the experimental data was reached if exclusively 1:1 stoichiometry was considered, whereas consideration of 2:1 (Lig–RNA) stoichiometries led to the divergence of the fit. Therefore, 1:1 stoichiometry was taken as preferred and further calculation was performed for this binding model, yielding the binding constant value of $K = (1.4 \pm 0.1) \times 10^5$ M^{-1} for the **2a**–RNA complex.

For derivative **1d**, the determination of the binding constant by spectrophotometric titration was not possible due to aggregation of the compound upon addition of RNA.

4. Biological studies

Well-plate screening

The oligonucleotides used for the well-plate screening were purchased from *Biomers.net GmbH* (Ulm, Germany).

- CAG RNA motif: 5'-GCAGCAGCUUCGGCAGCAGC-3'
- CUG RNA motif: 5'-GGUCGCUGCUGCACGAAAGUGCUGCUGCGACC-3'
- HIV-1 TAR RNA: 5'-GGCAGAUCUGAGCCUGGGAGCUCUCUGCC-3'
- HIV-1 RRE-IIB RNA: 5'-GGUCUGGGCGCAGCGCAAGCUGACGGUACAGGCC-3'

Prior to measurements, the RNA oligonucleotides were annealed by heating the RNA stock solutions to 95 °C followed by slow cooling down to room temperature overnight. To ensure the reproducibility, each measurement was repeated at least three times, the repeat experiments gave values within 20%.

RNA pull-down

HTT exon 1 with 51 CAG-repeat length was PCR amplified with the GoTaq® Green Master Mix (Promega) using a plasmid as template (HTT exon1 with 51 CAG repeats cloned into the multiple cloning site of pEGFP-C1 (primers: 5'-CCAAGCTTCTAATACGACTCACTATAGGGAGAATGGCGGACCCTGGAAAAGCTGATGAAGG-3' and 5'-GGTCGGTGCAGCGGCTCCTCAGC-3'). The forward primer contains the T7 phage promotor sequence to allow in vitro RNA synthesis by T7 RNA polymerase with the T7 RiboMAX™ Express Large Scale RNA Production System (Promega) and addition 0.5 mM biotin-UTP (Thermo Fisher Scientific, AM8450). Biotinylated RNA was purified and folded in RNA structure buffer [10 mM Tris (pH 7), 100 mM KCl, 10 mM MgCl2] by incubation at 72 °C, 10 min, followed by cooling to room temperature. Forty microliters of Streptavidin-Agarose Beads (SIGMA) per sample were coated with 80 pmol of biotinylated RNA in 500µl buffer D (20mM Tris pH 7.9, 20% glycerol, 0,1 M KCl, 0,2 mM EDTA, 0,5 mM DTT) for 30 min at room temperature. After washing the coated beads with buffer D, protein extract from HEK293T was added and the samples were incubated overnight at 4 °C. The beads were washed three times and resuspended in 20 µl 1 × SDS-PAGE sample buffer [50 mM Tris–Cl (pH 6.8), 100 mM DTT, 2% SDS, 0.1% bromophenol blue, 10% glycerol]. After denaturation at 95 °C for 10 min, the eluted proteins were analyzed by western blot using anti-MID1 antibodies (Abcam, Ab70770) to detect MID1.

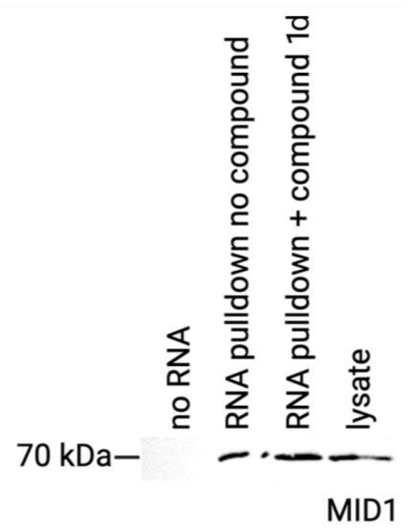

Figure S1. RNA-protein pull-down of MID1 with its target RNA HTT exon1 in the absence (w/o compound **1d**) or presence of the compound **1d** at a final concentration of 100 µM. RNA-bound proteins were analyzed on western blots detecting MID1. The expected band of approx. 70 kDa was detected in the RNA pulldown without **1d**, in the presence of **1d** and the cell lysate.

5. NMR data

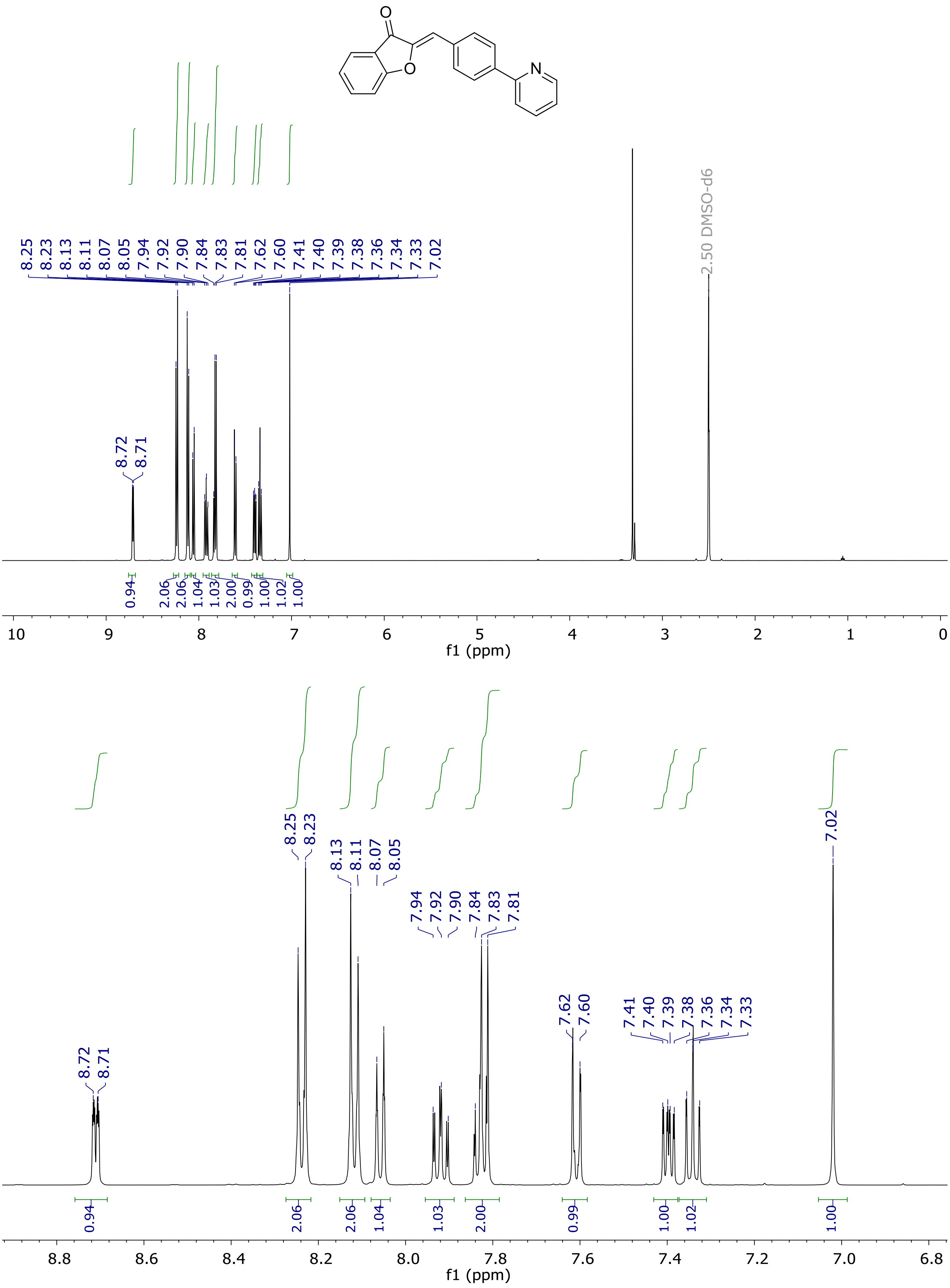

Figure S2. ^{1}H NMR spectrum of **1f** in DMSO-d_6 (top) with an expansion of the aromatic and double bond proton signals region (bottom).

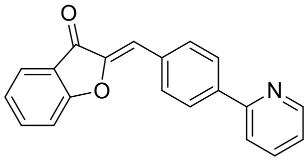

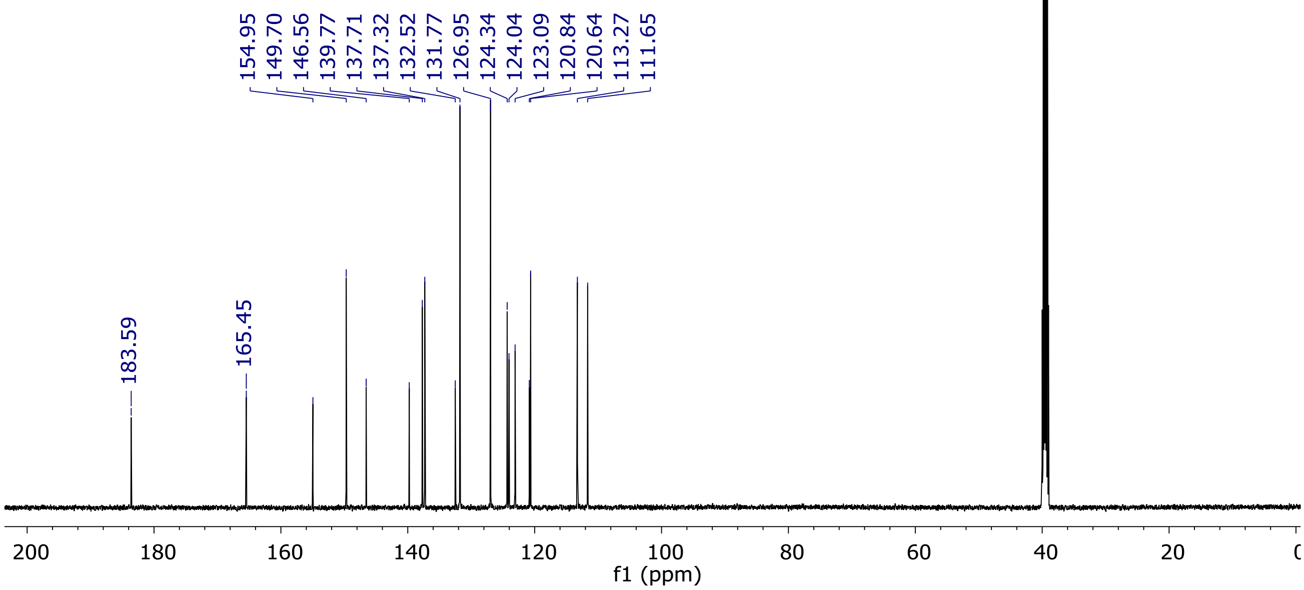

Figure S3. ^{13}C NMR spectrum of **1f** in DMSO-d_6.

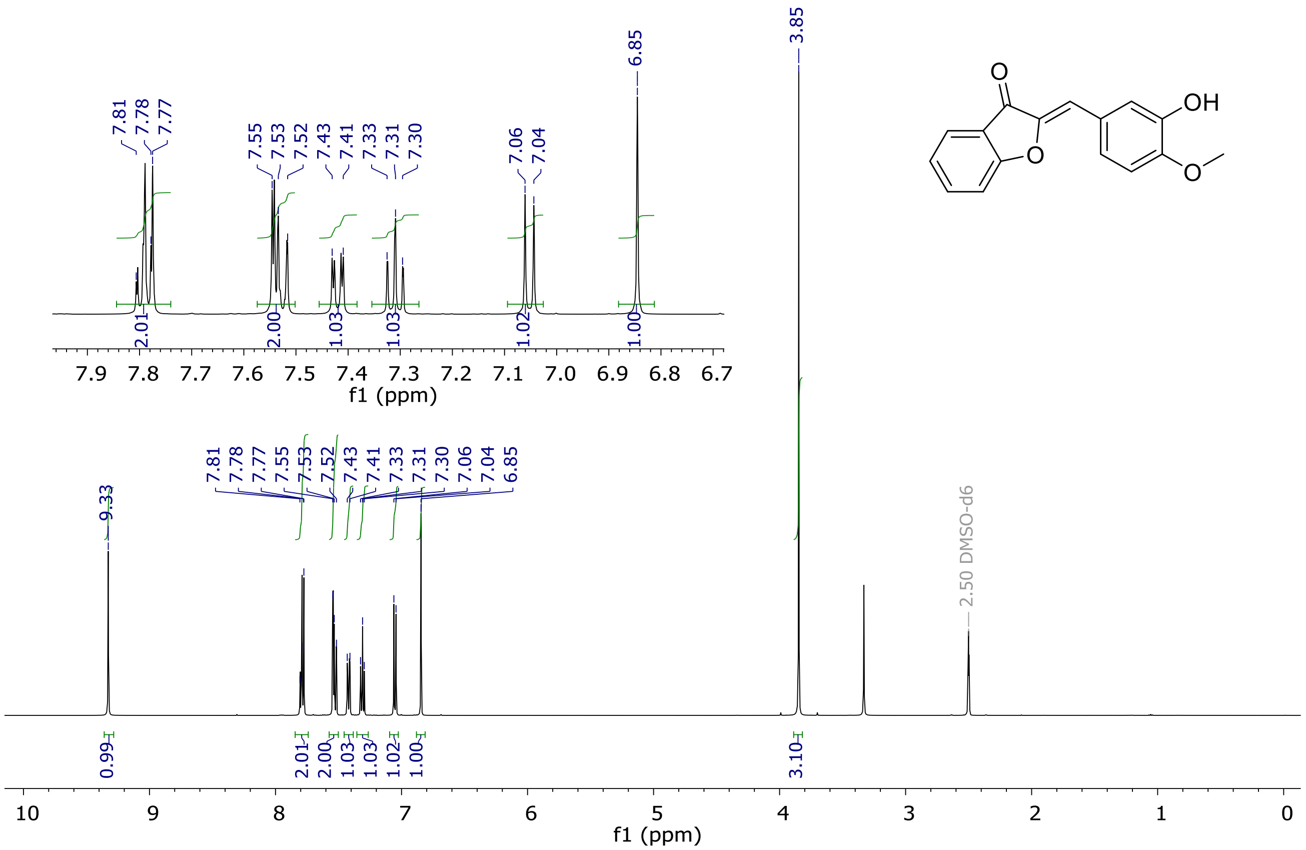

Figure S4. ^{1}H NMR spectrum of **1l** in DMSO-d_6 with an expansion of the aromatic and double bond proton signals region.

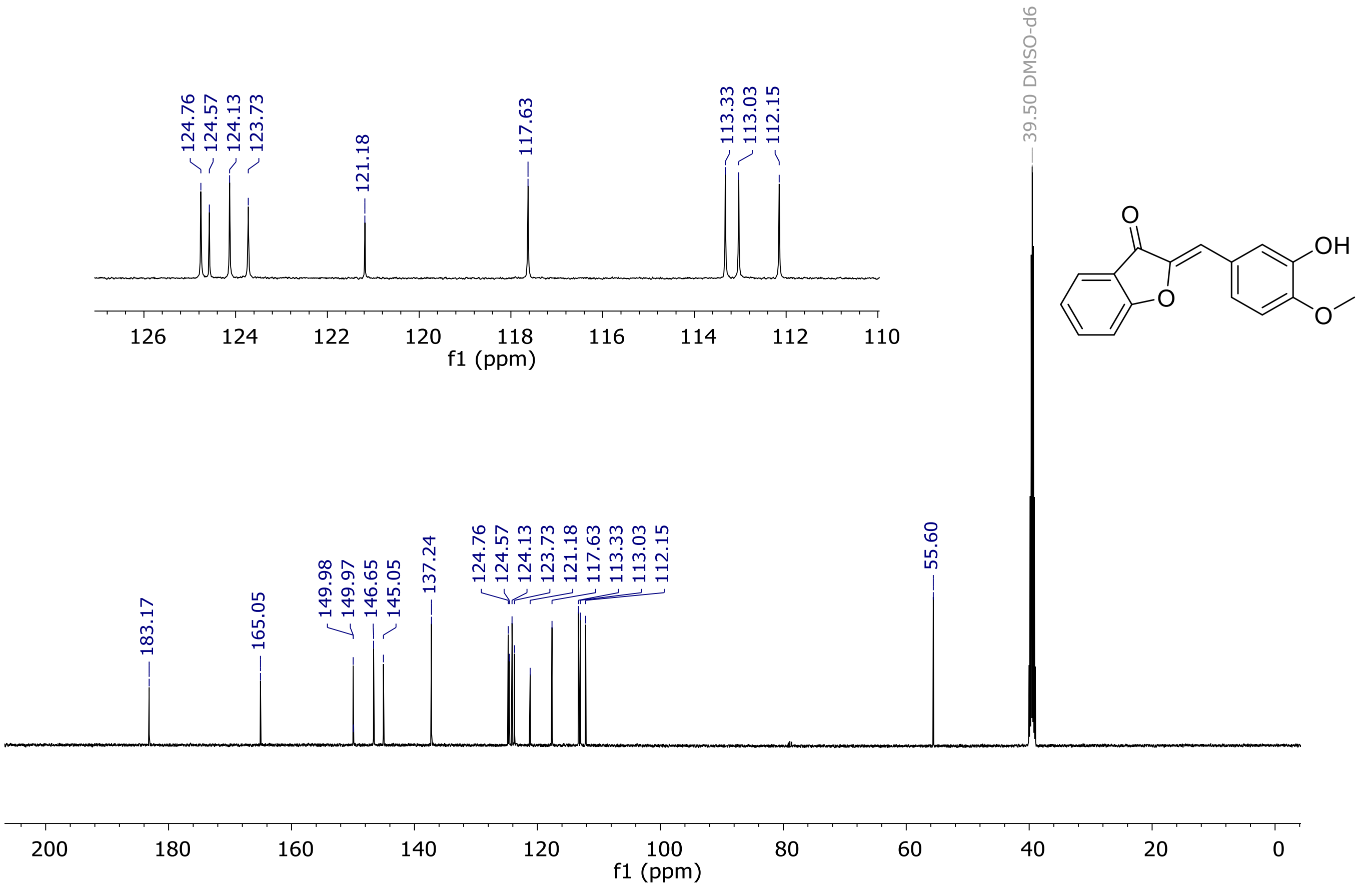

Figure S5. ^{13}C NMR spectrum of **1l** in DMSO-d_6.

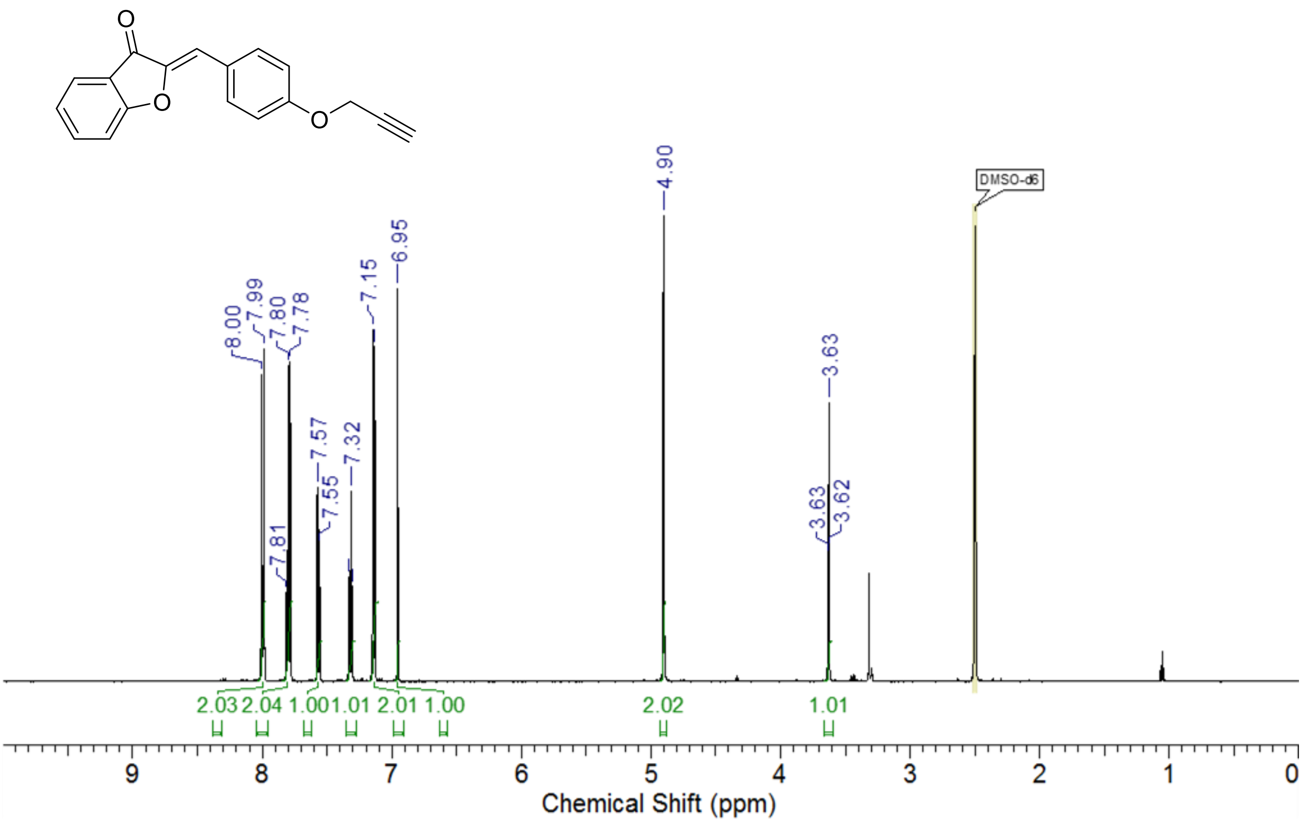

Figure S6. ^{1}H NMR spectrum of **1p** in DMSO-d_6 with an expansion of the aromatic and double bond proton signals region.

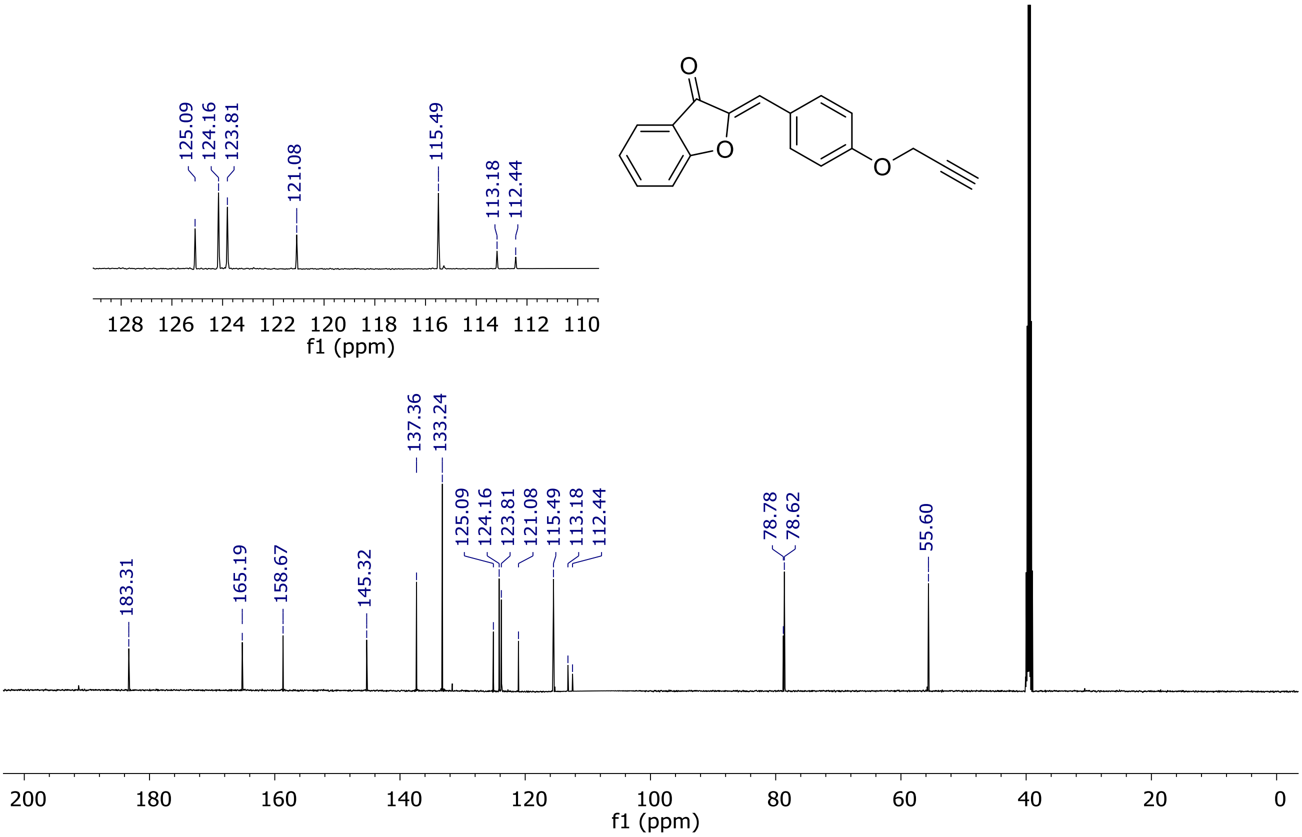

Figure S7. ^{13}C NMR spectrum of **1p** in DMSO-d_6 with an expansion of the aromatic and double bond proton signals region.

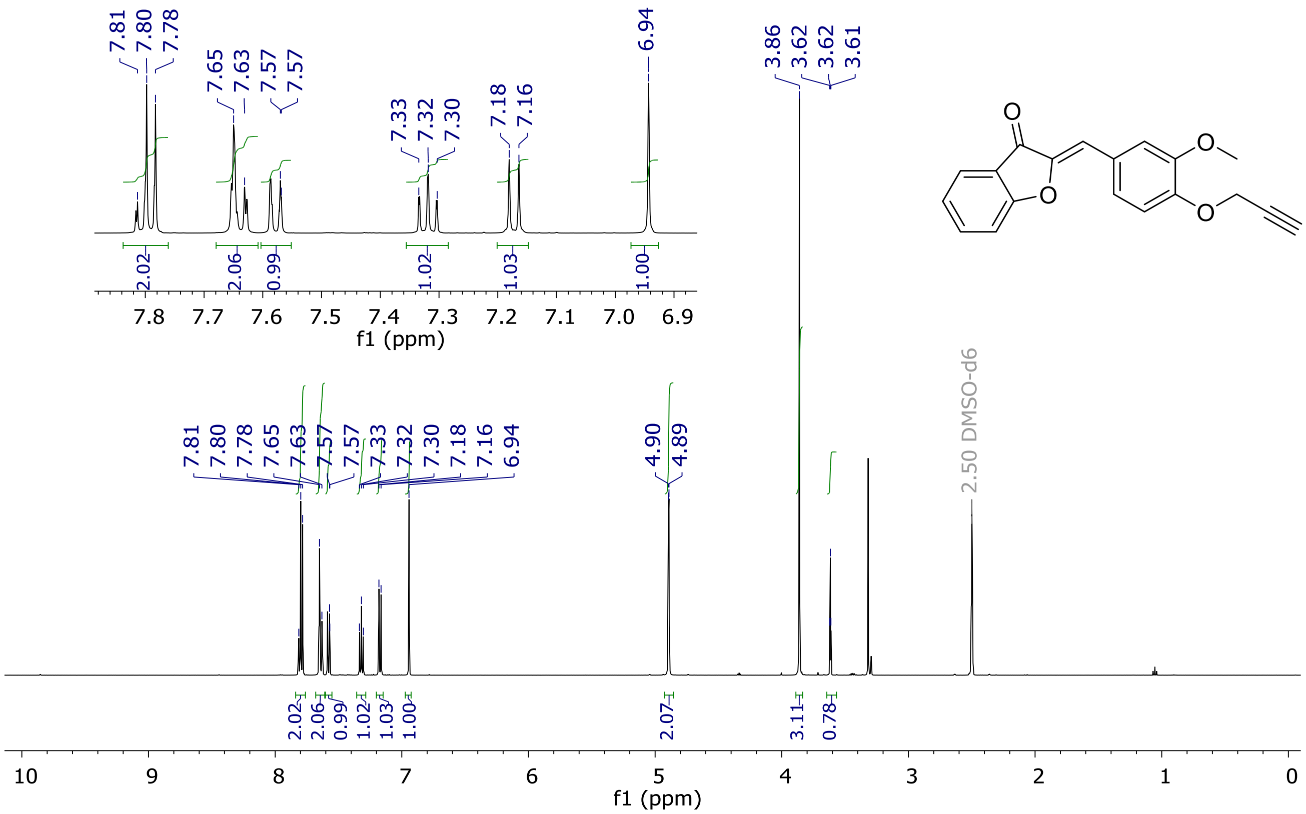

Figure S8. ^{1}H NMR spectrum of **1q** in DMSO-d_6 with an expansion of the aromatic and double bond proton signals region.

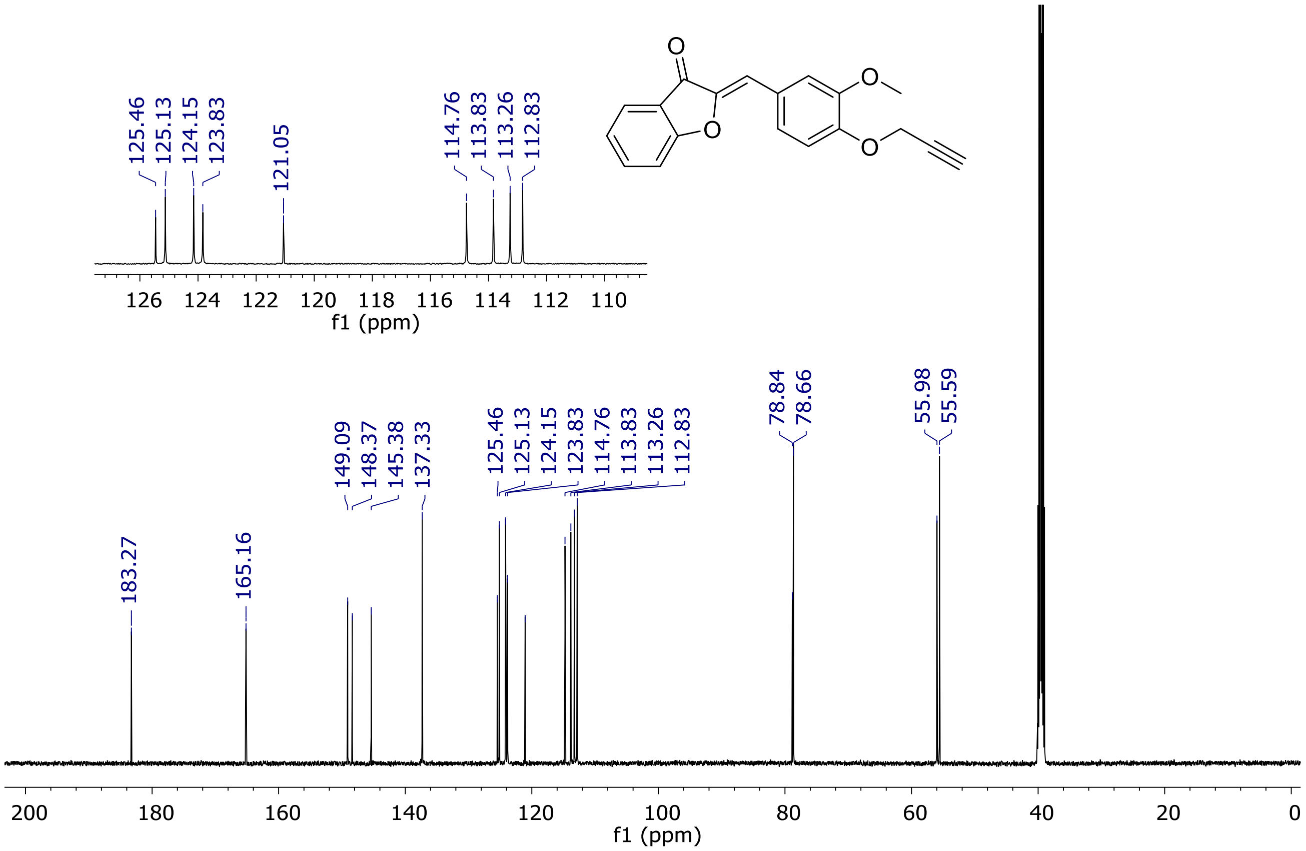

Figure S9. ^{13}C NMR spectrum of **1q** in DMSO-d_6 with an expansion of the aromatic and double bond proton signals region.

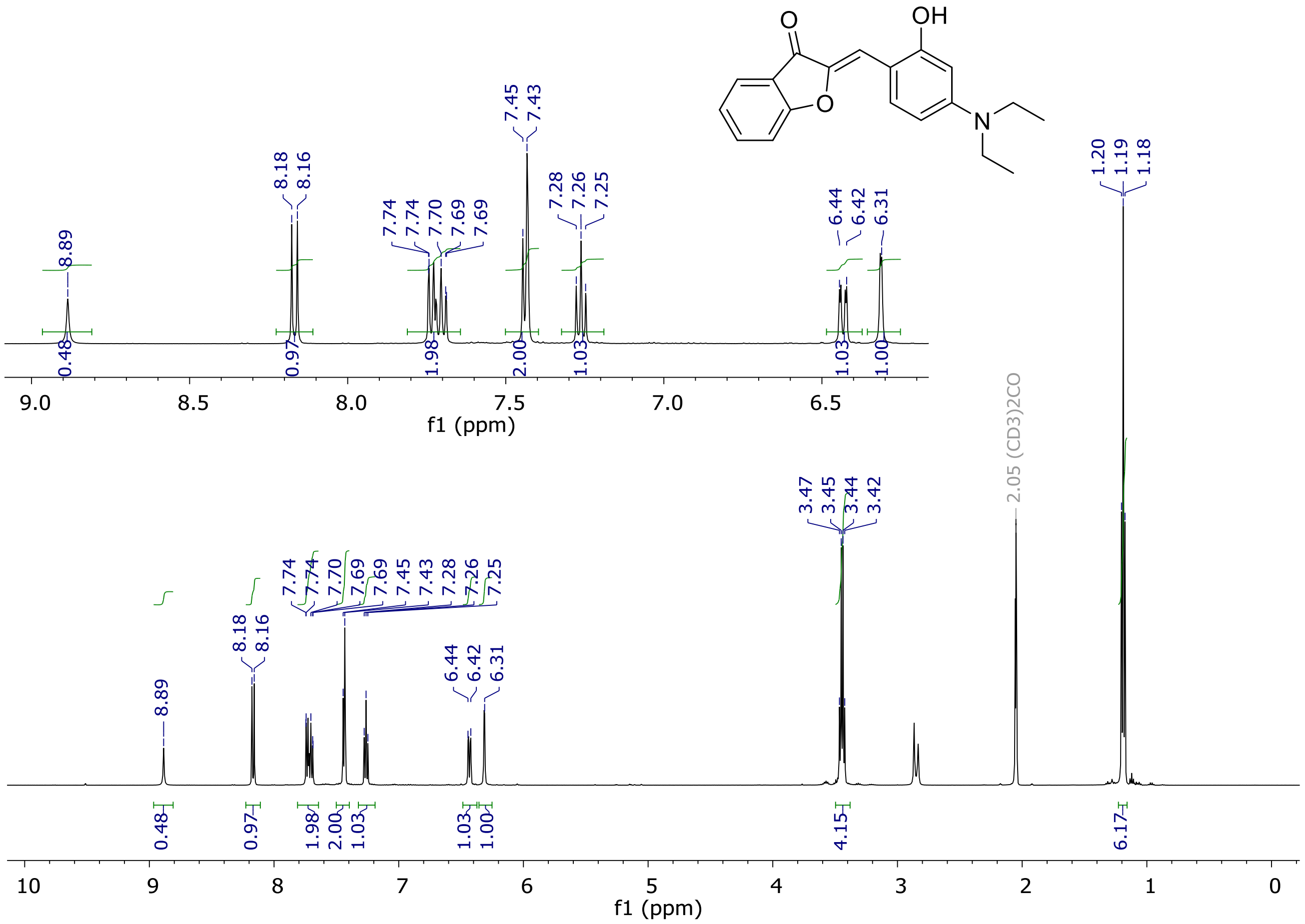

Figure S10. ^{1}H NMR spectrum of **1v** in acetone-d_6 with an expansion of the aromatic and double bond proton signals region.

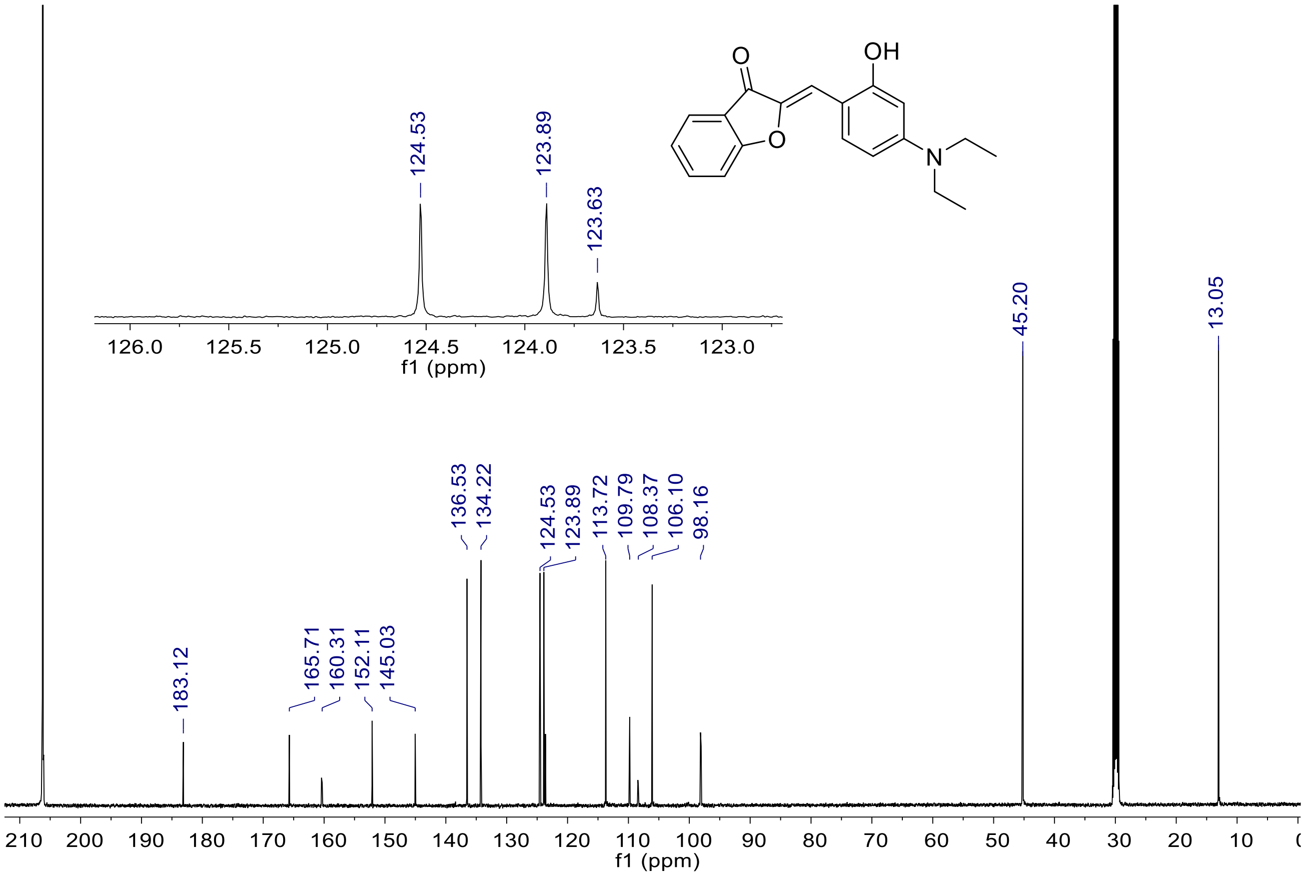

Figure S11. ^{13}C NMR spectrum of **1v** in acetone-d_6 with an expansion of the aromatic and double bond proton signals region.

6. Single-crystal X-ray crystallography data

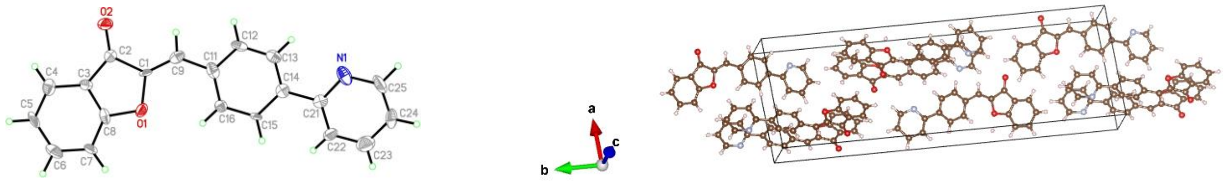

Figure S12. Structure (50 % thermal ellipsoids) and crystal packing of **1f** in the solid state.

Table S1. Crystal data and structure refinement for **1f**.

Identification code	db14	
Empirical formula	$C_{20}H_{13}NO_2$	
Formula weight	299.31	
Temperature	173(2) K	
Wavelength	0.71073 Å (Mo K_{α})	
Crystal system	Orthorhombic	
Space group	$Pna2_1$ (no. 33)	
Unit cell dimensions	a = 11.1186(11) Å	α = 90°.
	b = 33.698(4) Å	β = 90°.
	c = 3.8243(4) Å	γ = 90°.
Volume	1432.9(3) $Å^3$	
Z	4	
Density (calculated)	1.387 Mg/m^3	
Absorption coefficient	0.090 mm^{-1}	
F(000)	624	
Crystal colour, shape	light yellow needle	
Crystal size	0.090 x 0.010 x 0.010 mm^3	
Theta range for data collection	3.535 to 25.763°.	
Index ranges	$-13 \leq h \leq 12, -40 \leq k \leq 40, -4 \leq l \leq 4$	
Reflections collected	5933	
Independent reflections	2681 [R(int) = 0.1671]	
Completeness to theta = 25.000°	99.4 %	
Absorption correction	Semi-empirical from equivalents	
Max. and min. transmission	1.000 and 0.386	
Refinement method	Full-matrix least-squares on F^2	
Data / restraints / parameters	2681 / 1 / 208	
Goodness-of-fit on F2	1.227	
Final R indices [I>2sigma(I)]	R_1 = 0.1555, wR_2 = 0.2843	
R indices (all data)	R_1 = 0.2692, wR_2 = 0.3433	
Absolute structure parameter	0.3(10)	
Largest diff. peak and hole	0.436 and -0.513 e.$Å^{-3}$	

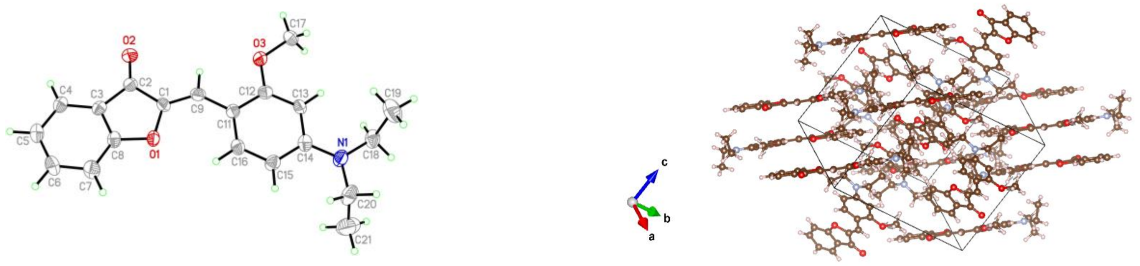

Figure S13. Structure (50 % thermal ellipsoids) and crystal packing of **1w** in the solid state.

Table S2. Crystal data and structure refinement for **1w**.

Identification code	db8	
Empirical formula	$C_{20}H_{21}NO_3$	
Formula weight	323.38	
Temperature	173(2) K	
Wavelength	0.71073 Å (Mo K_α)	
Crystal system	Monoclinic	
Space group	$P2_1/n$ (no. 14)	
Unit cell dimensions	a = 8.6748(8) Å	α = 90°.
	b = 16.7658(12) Å	β = 98.695(8)°.
	c = 11.7777(11) Å	γ = 90°.
Volume	1693.3(3) Å^3	
Z	4	
Density (calculated)	1.269 Mg/m^3	
Absorption coefficient	0.085 mm^{-1}	
F(000)	688	
Crystal colour, shape	red plate	
Crystal size	0.170 x 0.140 x 0.020 mm^3	
Theta range for data collection	3.382 to 25.027°.	
Index ranges	$-10 \leq h \leq 10$, $-18 \leq k \leq 19$, $-10 \leq l \leq 14$	
Reflections collected	6966	
Independent reflections	2967 [R(int) = 0.0487]	
Completeness to theta = 25.000°	99.4 %	
Absorption correction	Semi-empirical from equivalents	
Max. and min. transmission	1.000 and 0.743	
Refinement method	Full-matrix least-squares on F^2	
Data / restraints / parameters	2967 / 0 / 218	
Goodness-of-fit on F2	1.392	
Final R indices [I>2sigma(I)]	R_1 = 0.0979, wR_2 = 0.1466	
R indices (all data)	R_1 = 0.1330, wR_2 = 0.1567	
Largest diff. peak and hole	0.220 and -0.240 e.Å^{-3}	

7. References

[S1] Pelter, R. S. Ward, H. G. Heller, *J. Chem. Soc. Perkin Trans. 1*, **1979**, 328.

[S2] Stoe & Cie, X-AREA. Diffractometer control program system. Stoe & Cie, Darmstadt, Germany, **2002**.

[S3] G. M. Sheldrick, *Acta Crystallogr. Sect. A*, **2008**, *64*, 112.

[S4] T. A. Geissman, J. B. Harborne, *J. Am. Chem. Soc*., **1955**, *77*, 4622.

[S5] W. Zhao, J. Sun, H. Xiang, Y. Zeng, X. Li, H. Xiao, D. Chen, R. Ma, *Bioorg. Med. Chem.,* **2011**, *19*, 3192.

[S6] S. Okombi, D. Rival, S. Bonnet, A.-M. Mariotte, E. Perrier, A. Boumendjel, *J. Med. Chem.,* **2006**, *49*, 329.
